# Ammonia induces calpain-dependent cleavage of CRMP-2 during neurite degeneration in primary cultured neurons

**DOI:** 10.18632/aging.102053

**Published:** 2019-07-06

**Authors:** Zhenbin Cai, Xiaonan Zhu, Guowei Zhang, Fengming Wu, Hongsheng Lin, Minghui Tan

**Affiliations:** 1 Department of Orthopedics, The First Affiliated Hospital of Jinan University, Guangzhou, China; 2 Department of Anatomy, Medical College of Jinan University, Guangzhou, China

**Keywords:** ammonia, neurite degeneration, CRMP-2, calpain, GSK-3

## Abstract

Hyperammonemia in the CNS induces irreversible damages to neurons due to ultimate cell loss. Neurite degeneration, a primary event that leads to neuronal cell death, remains less elucidated especially in hyperammonemia circumstances. Here, we found that the administration of ammonia induced neurite degeneration in cultured cerebellar granule neurons. The resulting altered neuronal morphology, rupture of neurites, and disassembly of the cytoskeleton led to cell death. Calcein and Fluo-4 staining revealed that ammonia induced intracellular calcium dysregulation. Subsequently activated calpain cleaved CRMP-2, a microtubule assembly protein. Pharmacologically inhibition of calpain, but not caspases or GSK-3, suppressed the cleavage of CRMP-2 and reversed neurite degeneration under ammonia treatment. Exposure to ammonia decreased whereas inhibition of calpain restored the amplitude and frequency of miniature excitatory postsynaptic currents. These data suggest a mechanism by which elevated ammonia level may induce neuronal dysfunction via abnormal calcium influx and calpain-dependent CRMP-2 cleavage, leading to abnormal synaptic transmission, cytoskeletal collapse, and neurite degeneration.

## INTRODUCTION

Hyperammonemia is the critical factor contributed to the pathogenesis of several neurodegenerative disorders, such as hepatic encephalopathy (HE) and hyperammonemia (HA) [1, 2]. In addition, increased ammonium level in the brain is observed with inherited defects of the urea cycle [3], congestive heart failure [4], transient hyperammonemia of newborns [5] and other dysfunctions. The highest concentration of ammonium 15.5 mM has been reported in brain abscesses. Ammonia crosses the blood-brain barrier and induces astrocyte swelling and oxidative stress, which contribute to neuronal dysfunction [6]. High concentration of ammonia not only induces the dysfunction of astrocytes [7] but also directly leads to cell death of cultured neurons [8]. For example, ammonia induces apoptosis of cultured hippocampal neurons by altering BAD dephosphorylation [9] and activating NMDA receptors [8, 10], which may result in a massive influx of calcium ions [11, 12]. Exploring the precise mechanisms by ammonia-induced neuronal cell loss helps to identify new therapeutic targets.

One process that leads to neuronal apoptosis and death is neurite degeneration [13], an early event in many neurological conditions, including Parkinson’s disease, Alzheimer’s disease, and amyotrophic lateral sclerosis [14–16]. These and various other neurodegenerative diseases are also associated with altered function of collapsin response mediator protein 2 (CRMP-2) [17, 18]. CRMP-2 is a member of a family of cytoplasmic proteins enriched in developing and adult nervous systems [19–21] that are important for cell migration, neuronal polarity and neurite extension, and axonal guidance and regeneration [22, 23]. Members of the CRMP family act as microtubule-associated proteins targeting cytoskeletal microtubules and microfilaments [24, 25]. CRMP-2 is enriched in distal parts of growing neurites, where it binds to and helps transport tubulin dimers and actin filaments to facilitate cytoskeletal assembly [26–28]. Phosphorylation regulation by upstream kinases, such as GSK-3, Cdk5 [22] and Rho [29] leads to the inactivation of CRMP-2 and the dissociation from the cytoskeleton. Apart from phosphorylation regulation, CRMP-2 can be cleaved and inhibited by calpain during neurite degeneration [30, 31]. However, how these regulations of CRMP-2 participate in hyperammonemia scenario remain to be explored.

To elucidate whether ammonia may impact neuronal morphology and the mechanism by which this may occur, cultured cerebellar granule neurons were treated with ammonia and analyzed for changes in morphology and calcium-dependent processes. We found that ammonia treatment induced calcium influx and calpain activation, leading to the cleavage of CRMP-2 and ultimately neurite degeneration and cell death.

## RESULTS

### Ammonia induces neurite degeneration in cultured cerebellar neurons

**Figure 1:**
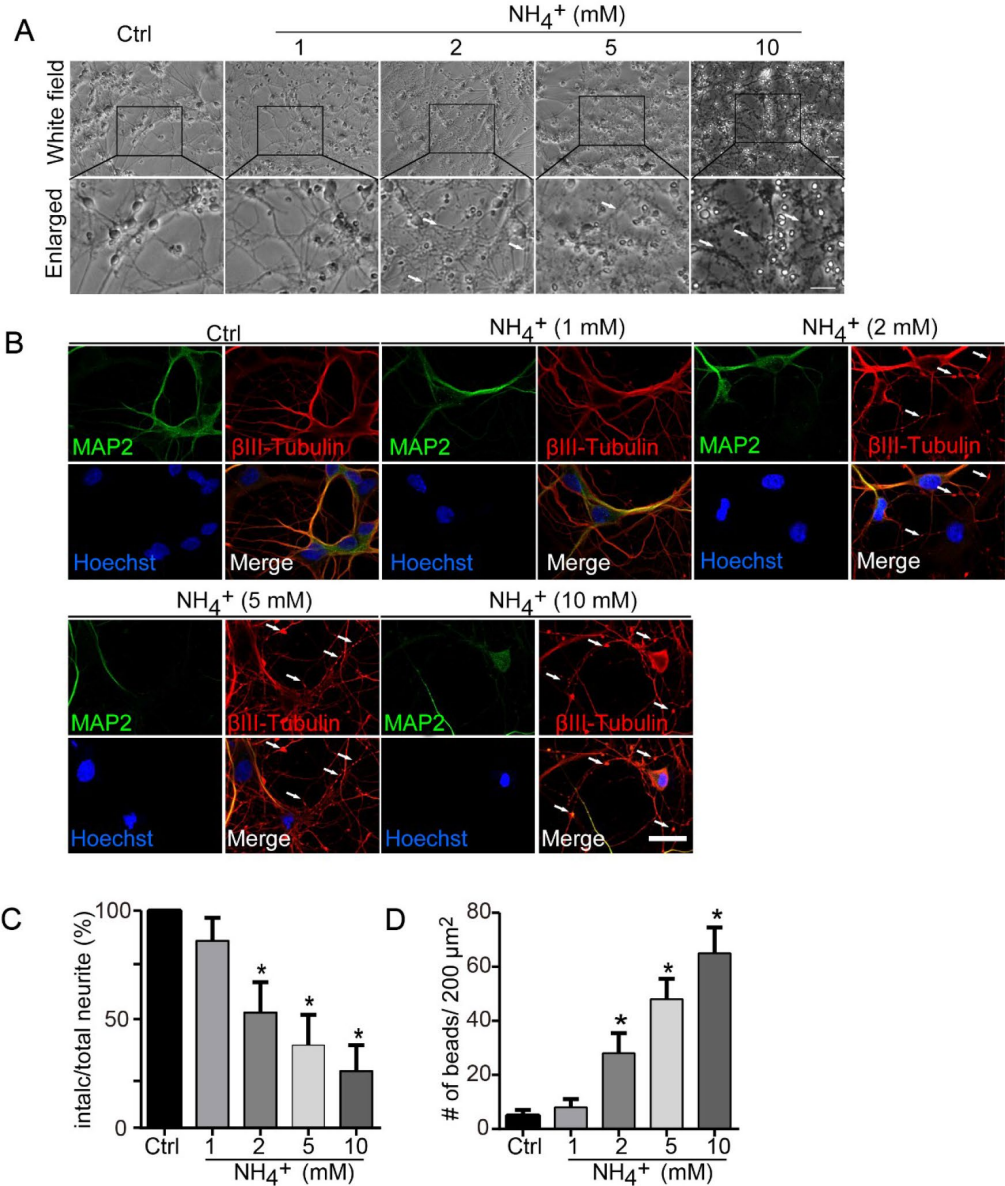
**Ammonia induces axonal degeneration of cultured neurons.** (**A**) Axonal beading and fragmentation in the course of degeneration induced by ammonia (1, 2, 5, and 10 mM for 24 h) in cerebellar granule neurons (CGNs) cultured for DIV 7. Morphological changes of neurons were monitored with phase-contrast microscopy, and representative and enlarged images were shown. White arrows show the beading formation. Scale bar, 50 μm. (**B**) Same treated neurons were subjected to immunocytochemistry with MAP2 staining (Green signal) for dendrites and βIII-Tubulin (red signal) for neurites. Hoechst staining shows the nucleus of neurons. Whiter arrows show the beading formation along the axonal shafts. Dendrites revealed by MAP2 showed no varicosities. Scale bar, 20 μm. Quantitative measurements of neurite fragmentation (**C**) and axonal beading (**D**). Relative percentages in the control group (Ctrl) were set at 100% in (**C**).* *p* < 0.05 vs. Ctrl.

Primary cerebellar granule neurons cultured for 7 days *in vitro* were treated with 1, 2, 5, or 10 mM ammonia for 24 h and observed by microscopy for morphological changes. The treatments induced neurite degeneration, observed as swollen puncta along axons above 2 mM ammonia, neurite beading and fragmentation above 5 mM, and neurons becoming transparent, indicating cell death, at 10 mM (Figure 1A). To identify the affected neurites, immunofluorescence staining with MAP2 for dendrites and βIII-tubulin for axons were performed. As shown in Figure 1B, the dendrite shafts showed no significant morphological changes, extending smoothly in each ammonia-treated group as control group. However, the varicosities and number of beading increased in Tubulin-positive neurites that did not colocalize with MAP2, in an ammonia-concentration dependent manner. These changes were quantified by measurements of the proportions of intact neurites remaining (Figure 1C) and the density of neurite beads along neurites (Figure 1D). The data suggest that ammonia administration mainly affect the morphological changes of axons but not dendrites.

**Figure 2:**
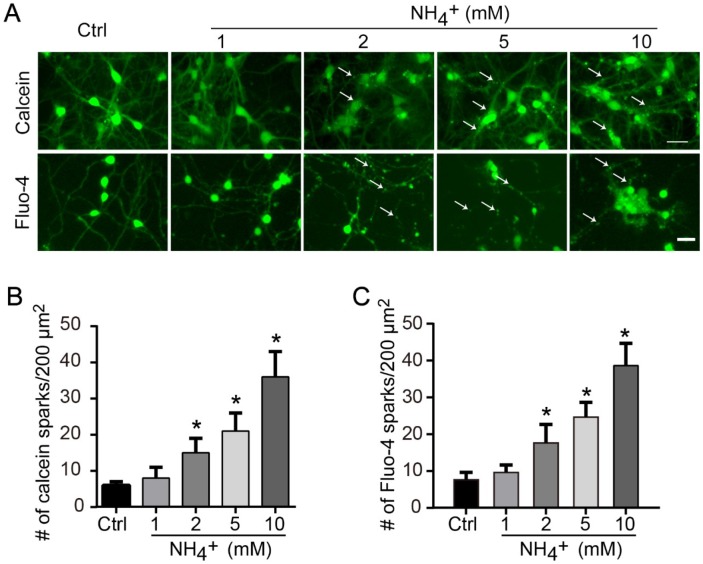
**Ammonia treatment induces locally upregulation of calcium concentration.** (**A**) For intracellular calcium staining, 2 μM calcein-AM or 2 μM Fluo-4 were added to culture medium for 30 min before image capture. Quantitative data of number of calcein puncta (**B**) or Fluo-4 sparks (**C**) indicate high concentrations of calcium. Data are means ± SEs from at least three independent experiments; * *p* < 0.05 vs. Ctrl. Scale bar, 20 μm.

To assess whether these changes involve ammonia-induced calcium dysfunction, live neurons were stained with the membrane-permeable dye calcein-AM. Ammonia-treated cells exhibited fluorescent puncta within the cell body and along the neurites, indicative of high local concentrations of calcium (Figure 2A, upper panel). The numbers of these puncta increased in an ammonia concentration-dependent manner (Figure 2B). Because calcein staining is very pH sensitive and hard to reveal puncta from lipid vesicles, we also applied Fluo-4 staining (Figure 2A, lower panel). The statistical results of Fluo-4 staining (Figure 2D) showed the same trend as calcein (Figure 2C). These data indicate that ammonia induces neurite degeneration, cytoskeletal collapse, and finally neuronal death, which may result from the increase of local calcium concentration.

### Ammonia induces calpain-mediated CRMP-2 cleavage

**Figure 3:**
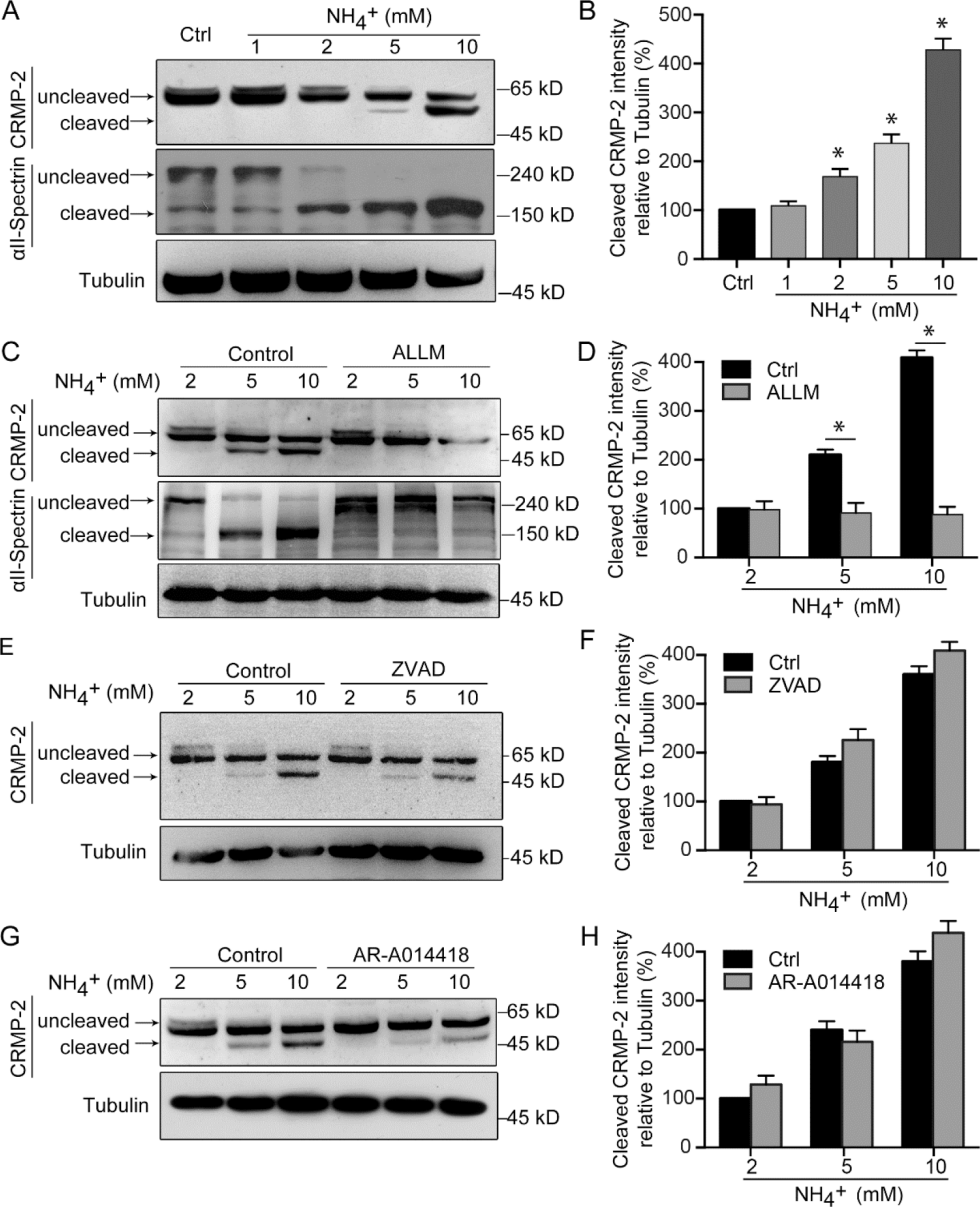
**CRMP-2 is cleaved after ammonia treatment, which is mediated by calpain.** Cultured cerebellar granule cells were treated as in Figure 1, and then neuronal lysates were subjected to western blotting with antibodies against CRMP-2 and αII-Spectrin (**A**). (**B**) Quantification of intensity bands for cleaved CRMP-2 relative to that for tubulin. Relative value in control group was set to 100%. Western blotting analyses of cultured cerebellar granule cells treated along with calpain inhibitor ALLM (10 μM) (**C** and **D**), caspase inhibitor ZVAD (20 μM) (**E** and **F**), and GSK-3 inhibitor AR-A014418 (10 μM) (**G** and **H**). Data are means ± SEs from at least three independent experiments; * *p* < 0.05 vs. Ctrl.

As the dysregulation of intracellular calcium levels can alter the activity of a variety of calcium-dependent enzymes, such as calpain [32], we sought to assess if ammonia treatment induces the cleavage of proteins important for regulating cytoskeletal structure. We focused on CRMP-2, which is known to be cleaved by calpain [30, 31, 33–36]. Western blot analysis of cerebellar granule neurons revealed increasing amounts of cleaved CRMP-2 with treatments of increasing concentrations of ammonia (Figure 3A and 3B). To determine if the cleavage of CRMP-2 was a result of enzyme activity, we treated cells with several inhibitors in addition to ammonia. The western blot analysis revealed that the cleavage of CRMP-2 in ammonia-treated cells was significantly suppressed when calpain was inhibited by ALLM (Figure 3C and 3D) but not when caspases were inhibited by ZVAD (Figure 3E and 3F), confirming that ammonia-induced CRMP-2 cleavage is calpain dependent. Moreover, Spectrin is a well-characterized substrate for calpain [37, 38]. We found that ammonia administration significantly induced the cleavage of Spectrin, with the upregulation of 150 kD cleaved band, in an ammonia-concentration dependent pattern (Figure 3A, lower panel). The cleavage also can be inhibited by the application of calpain inhibitor ALLM (Figure 3C). We next sought to determine whether the cleavage of CRMP-2 is dependent on its activity. As microtubule binding activity of CRMP-2 can be inhibited by phosphorylation with GSK-3 [22, 39], we cotreated cells with ammonia and a GSK-3-specific inhibitor, AR-A014418. The results showed that inhibition of GSK-3 did not significantly impact ammonia-induced CRMP-2 cleavage (Figure 3G and 3H). Altogether, these data indicate that exposure to ammonia induces calpain-dependent cleavage of CRMP-2 in cerebellar granule neurons.

### Inhibition of calpain prevents ammonia-induced neuronal degeneration and rescues synaptic dysfunction

**Figure 4:**
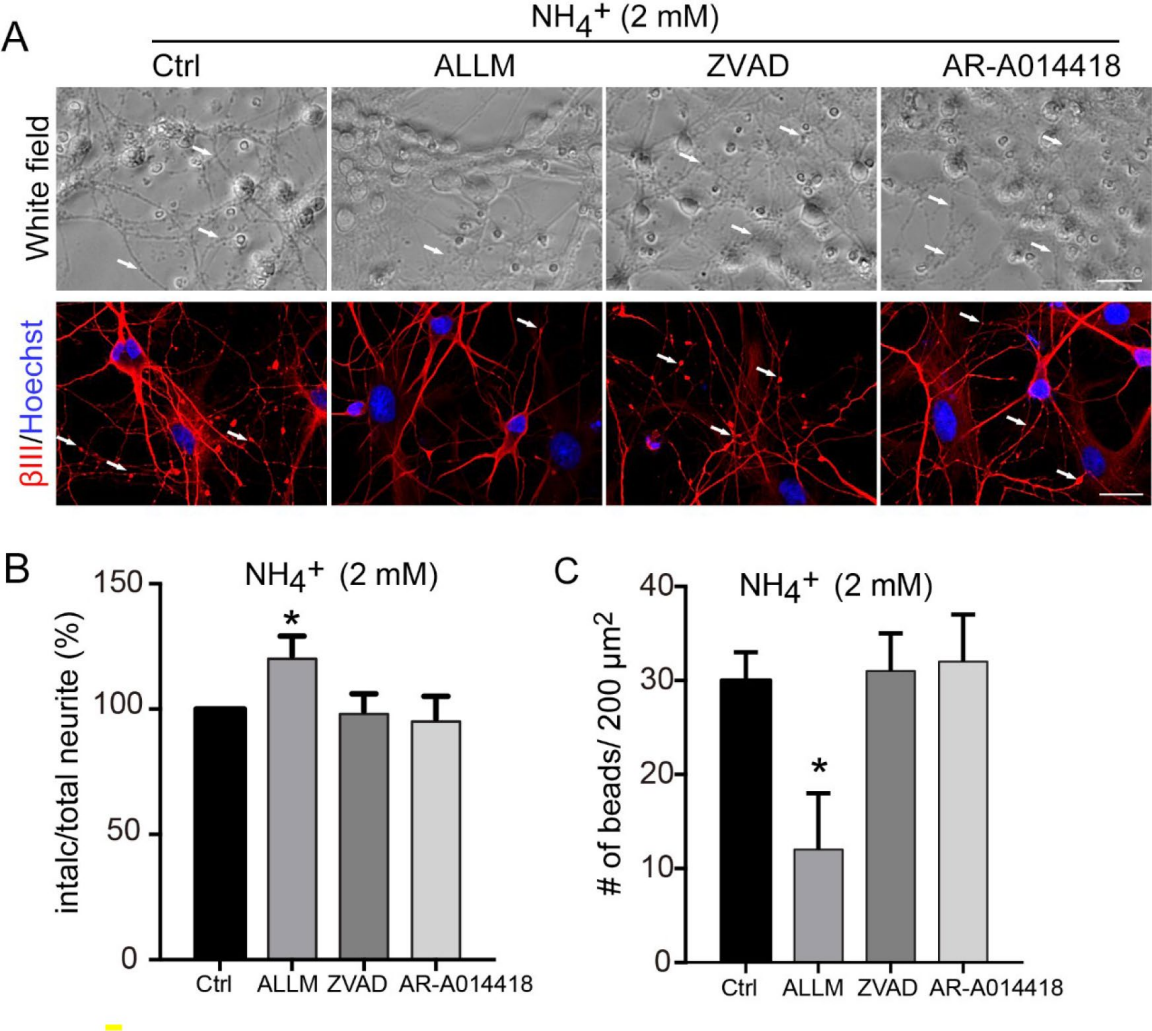
**Axonal degeneration induced by ammonia is mediated by calpain.** (**A**) Cultured cerebellar granule cells were treated with 2 mM ammonia for 24 h with or without ALLM, ZVAD, or AR-A014418, and observed by microscopy. Neurons were subjected to immunocytochemistry with βIII-Tubulin (βIII) and Hoechst for nucleus staining. White arrows show the beading puncta. Neurite fragmentation (**B**) and axonal beading (**C**) were quantified as in Figure 1. Data are means ± SEs from at least three independent experiments; * *p* < 0.05 vs. Ctrl. Scale bar, 20 μm.

As inhibition of calpain prevented ammonia-induced CMRP-2 cleavage, we next assessed whether it would also prevent ammonia-induced morphological changes. Treatment of cells with the calpain inhibitor ALLM prevented the appearance of neurite degeneration (Figure 4A), increased the proportion of intact neurites (Figure 4B) and reduced bead formation (Figure 4C), comparing to those in cells treated with ammonia only. Treatments with ZVAD and AR-A014418 had no effect, consistent with their inability to block ammonia-induced CRMP-2 cleavage.

**Figure 5:**
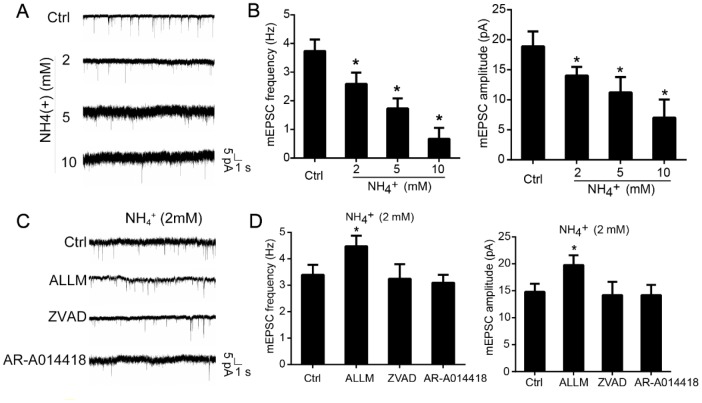
**Inhibition of calpain prevents ammonia-induced impairment of synaptic activity.** Cultured cerebellar granule cells were treated with ammonia (0, 2, 5, or 10 mM) for 24 h and subjected to patch-clamp electrophysiological recording. Representative tracings (**A**) and quantification of frequencies and amplitudes (**B**) of mEPSCs. Representative tracings (**C**) and quantification of frequencies and amplitudes (**D**) of mEPSCs in neurons treated with 2 mM ammonia for 24 h with or without ALLM, ZVAD, or AR-A014418. Data are means ± SEs; *n* =15 cells from three cultures from seven animals; * *p* < 0.05 vs. Ctrl.

Additionally, we assessed whether ammonia would impact synaptic activity. Patch-clamp recordings of cultured cerebellar granule cells revealed that treatment with ammonia destabilized baseline activity (Figure 5A). Moreover, ammonia dose-dependently decreased the amplitude and frequency of miniature excitatory postsynaptic currents (mEPSCs, Figure 5B). Notably, these effects were blocked by the inhibition of calpain with ALLM, which resulted in increased frequency and amplitude of mEPSCs (Figure 5C and 5D). Not surprisingly, ZVAD and AR-A014418 had no effect. Altogether, these data suggest that calpain activation upon exposure to ammonia induces the morphological and physiological dysfunction in cultured neurons.

## DISCUSSION

The results of this study show that ammonia directly induces neurite degeneration and morphological and functional deficits in cultured neurons. Specifically, ammonia produces calcium dysregulation, calpain activation, CRMP-2 cleavage, cytoskeleton collapse and alterations in mEPSCs. These effects represent a potential mechanism for the pathogenesis of hyperammonemia, a condition in which the excessive accumulation of ammonia in the brain is considered the primary inducer [40, 41]. As ammonia is shown to induce astrocytic swelling and to disturb normal astrocytic neuronal interactions and neural transmission [42], astrocytes have been considered the primary target of ammonia toxicity, with secondary effects on neighboring neurons. The evidence here introduces neurons as a primary target of ammonia and proposes a potential mechanism leading to neurological dysfunction.

Patients with liver cirrhosis develop hepatic encephalopathy with mild cognitive impairment, attention deficits and psychomotor, where hyperammonemia plays synergistic roles [43]. Hyperammonemia induces cognitive impairment by directly altering glutamatergic and GABAergic neurotransmission dysfunctions [44–46]. For example, ammonia would prevent activation of NMDA receptors in rat cerebellar neuronal cultures [44], chronic hyperammonemia would impair the extracellular glutamate and glutamate-nitric oxide-cyclic GMP pathway in cerebellar neurons in culture and in the rat *in vivo* [45, 47], hyperammonemia alters membrane expression of GluA1 and GluA2 subunits of AMPA receptors in hippocampus with altered spatial memory in rats [48]. Chronic hyperammonemia induces inflammation that leads to cognitive impairment in rats [49]. Thus, altered neurotransmission is significantly observed in the cerebellum [50]. Here, in our current study, we observed that ammonia treatment markedly induced the impairment of mEPSCs in cultured cerebellar granule neurons, and inhibition of calpain/CRMP-2 signaling component would partially rescue the transmission dysfunction (Figure 5). Our results provide detailed signaling pathways supplementing the mechanisms of psychomotor dysfunction in patients with hyperammonemia.

Oxidative stress also plays an important role in ammonia neurotoxicity. High levels of ammonia increase reactive oxygen and nitrogen species in hyperammonemia models *in vivo*, as well as in cultured systems [51]. Antioxidant enzymes and lipid peroxidation in the brain are reduced and tyrosine nitration in astrocytes is increased in various ammonia-treated models [7, 52]. Protein tyrosine nitration, the oxidation of RNA, and the activation of Zn-dependent gene transcription in the brain are considered the main downstream consequences of oxidative stress, which account for HE symptoms [53, 54]. Moreover, low-grade cerebral edema in HE patients may result from astrocyte swelling when exposed to ammonia caused by increased intracellular concentrations of calcium [55] and later oxidative stress [56]. A recent study showed that ammonia also significantly induces reactive oxygen species in cerebellar granule cells and that pharmacological inhibition suppressed ammonia-induced damage by oxidative stress [57]. Accordingly, we found that ammonia treatment influences calcium distribution, which likely triggered the observed neurite beading and degeneration, and may have resulted in additional oxidative stress. Further investigations are needed to clarify the effect of ammonia on oxidation and reactive oxygen species production in neurons.

We demonstrate that ammonia alters calcium regulation and induces calpain-dependent cleavage of a protein regulating the cytoskeletal structure of neurons, namely, CRMP-2. However, we believe many other calcium-dependent kinases may also contribute to ammonia-induced neuronal damage. CRMP-2 facilitates tubulin assembly and promotes microtubule stability [58], and a disruption of this may retard axonal growth and result in axonal degeneration. CRMP-2 dissociates from microtubules following phosphorylation by GSK-3 [22] and Cdk5 [59], or after cleavage by calpain, thereby potentially greatly impacting axonal integrity and maintenance. We provide direct evidence of this, as the prevention of CRMP-2 cleavage by calpain inhibition blocked that degeneration of axons induced by ammonia. Additionally, calpain can be activated by NMDA administration and CaMKII, which also phosphorylates CRMP-2, resulting in axonal varicosities [60]. However, this phosphorylation preceded large-scale cleavage of CRMP-2, which was not suppressed by inhibitors of CaMKII, indicating that cleavage of CRMP-2 is phosphorylation-independent [60]. Our results are consistent with this, as inhibition of GSK-3 did not prevent CRMP-2 cleavage. Recent reports show that CRMP-2 interacts with calcium channel CaV2.2 to regulate neurotransmission [61, 62]. And Ckd5-mediated phosphorylation of CRMP-2 enhances the interaction with CaV2,2 [63]. Phosphorylation and SUMOylation coordinately regulate CRMP-2 activity to mediate neurotransmission [64, 65]. Thus, including cleavage, the post transcriptional modifications of CRMP-2 are important for its regulation under various pathophysiological conditions.

In summary, the results of this study demonstrate that high ammonia directly induces neurite degeneration via calpain-dependent cleavage of CRMP-2. Thus, modulation of the calpain/CRMP-2 pathway represents a potential avenue for future effective therapies for ammonia-induced neurite damage.

## MATERIALS AND METHODS

### Cell culture and treatment

Cerebellar granule cells were prepared from 7-day-old Sprague-Dawley rat pups as previously described [39, 66], producing cultures of high purity (> 95% granule neurons) [67]. Briefly, neurons were dissociated from freshly dissected cerebella by mechanical disruption in the presence of trypsin and DNase, and then seeded at a density of 1.5 × 10^6^ cells/ml in basal modified Eagle’s medium containing 10% fetal bovine serum (Gibco BRL, Gaithersburg, MD) and 25 mM KCl (Sigma-Aldrich, St Louis, MO), a concentration sufficient to induce membrane depolarization. For electrophysiological analyses, the cells were seeded on coverslips (Thermo Fisher Scientific, Rockford, IL).

All animal procedures were performed in strict accordance with the recommendations in the Guide for the Care and Use of Laboratory Animals of the National Institutes of Health. The protocol was approved by the Jinan University Institutional Animal Care and Use Committee. All efforts were made to minimize the suffering and number of animals used.

### Morphological assessments and calcium staining

Neurite degeneration was defined by beading (swellings along neurite) and fragmentation as previously described [60, 68, 69]. Briefly, cultured neurons were incubated by ammonia with or without pre-incubation of the calpain inhibitor (ALLM, 10 μM, Sigma), the pan-caspase inhibitor (ZVAD, 20 μM, Sigma) and the GSK-3 inhibitor (AR-A014418, 10 μM, Sigma) for 30 min, then neuronal morphology was evaluated. For calcium release indication, calcein-AM and Fluo-4 (all purchased from Thermo Fisher Scientific, Carlsbad, CA, USA) was applied to cultured neurons. Briefly, neurons grown on coverslips were incubated with calcium indicator 2 μM calcein-AM for 30 min at 37°C and viewed through using a fluorescent microscopy.

### Western blotting

Western blotting analyses were performed as described previously [70, 71]. Briefly, lysates were separated by SDS-polyacrylamide gel electrophoresis and electrophoretically transferred to polyvinylidene fluoride membranes. The membranes were blocked in Tris-buffered saline with 5% milk and 0.05% Tween 20 and probed with primary antibodies against CRMP-2 (Cell Signaling Technology, Danvers, MA) and tubulin (Sigma-Aldrich) at 4°C overnight. Appropriate horseradish peroxidase-conjugated secondary antibodies (Jackson Immuno-Research, West Grove, PA) were used for detection with enhanced chemiluminescence reagents (GE Healthcare, Chalfont St Giles, UK).

### Electrophysiology

Whole-cell patch-clamp recordings of miniature excitatory synaptic currents (mEPSCs) were obtained from neurons cultured for 10 days *in vitro* [72–74]. During the recordings, the cells were bathed in an external solution (pH 7.3) containing 128 mM NaCl, 5 mM KCl, 2 mM CaCl_2_, 1 mM MgCl_2_, 15 mM glucose, 20 mM HEPES, 1 mM tetrodotoxin, and 100 μM picrotoxin. Recording pipettes were filled with an intracellular solution containing 147 mM KCl, 5 mM Na_2_-phosphocreatine, 2 mM EGTA, 10 mM HEPES, 2 mM MgATP, and 0.3 mM Na_2_GTP. Recordings were performed at room temperature in voltage clamp mode, at a holding potential of −70 mV, using a Multiclamp 700 B amplifier (Molecular Devices, Sunnyvale, CA) and Clampex 10.5 software (Axon Instruments, Union City, CA). The series resistance was below 30 MΩ, and data were acquired at 10 kHz and filtered at 1 kHz. mEPSCs were analyzed using MiniAnalysis software (Synaptosoft, Decatur, GA, USA) from experiments performed at least three times.

### Immunocytochemistry

Immunocytochemistry was performed as previously described [39]. Briefly, CGNs cultured on coverslips after treatments were fixed with 4% paraformaldehyde and permeabilized with 0.1% Triton X-100 in Tris-buffered saline and blocked in 3% donkey serum. Then neurons on the coverslips were incubated with the mouse anti-MAP2 (Chemicon, Temecular, CA, USA) and rabbit anti-βIII tubulin (Cell Signaling Technology). Then secondary antibodies conjugated to Alexa Fluor 488 or 555 (Molecular Probes, Leiden, the Netherlands) were incubated to link the primary antibodies. The coverslips were mounted with Fluore-Gel II with DAPI (Electron Microscopy Sciences, Hatfield, PA, USA) and images were captured with a Carl Zeiss LSM 780 confocal microscope (Zeiss, Germany).

### Statistical analysis

The statistical significance of the differences between two groups was determined by Student’s *t*-tests, and comparisons between more than two groups were performed with one-way analyses of variance with Newman–Keuls *post hoc* tests. Data were presented as mean ± SE. A *p* value of < 0.05 was considered statistically significant.

## References

[1] Rahimi RS, Rockey DC. Hepatic Encephalopathy: Pharmacological Therapies Targeting Ammonia. Semin Liver Dis. 2016; 36:48–55. 10.1055/s-0036-1571298 26870932

[2] Parekh PJ, Balart LA. Ammonia and Its Role in the Pathogenesis of Hepatic Encephalopathy. Clin Liver Dis. 2015; 19:529–37. 10.1016/j.cld.2015.05.002 26195206

[3] Helman G, Pacheco-Colón I, Gropman AL. The urea cycle disorders. Semin Neurol. 2014; 34:341–49. 10.1055/s-0034-1386771 25192511

[4] Evans JM, Fazekas JF, Ticktin HE. Ammonia intoxication in a patient with congestive heart failure receiving ammonium chloride orally. N Engl J Med. 1956; 255:905–07. 10.1056/NEJM195611082551907 13369738

[5] Alfadhel M, Mutairi FA, Makhseed N, Jasmi FA, Al-Thihli K, Al-Jishi E, AlSayed M, Al-Hassnan ZN, Al-Murshedi F, Häberle J, Ben-Omran T. Guidelines for acute management of hyperammonemia in the Middle East region. Ther Clin Risk Manag. 2016; 12:479–87. 10.2147/TCRM.S93144 27099506 PMC4820220

[6] Reinehr R, Görg B, Becker S, Qvartskhava N, Bidmon HJ, Selbach O, Haas HL, Schliess F, Häussinger D. Hypoosmotic swelling and ammonia increase oxidative stress by NADPH oxidase in cultured astrocytes and vital brain slices. Glia. 2007; 55:758–71. 10.1002/glia.20504 17352382

[7] Widmer R, Kaiser B, Engels M, Jung T, Grune T. Hyperammonemia causes protein oxidation and enhanced proteasomal activity in response to mitochondria-mediated oxidative stress in rat primary astrocytes. Arch Biochem Biophys. 2007; 464:1–11. 10.1016/j.abb.2007.03.027 17475207

[8] Klejman A, Wegrzynowicz M, Szatmari EM, Mioduszewska B, Hetman M, Albrecht J. Mechanisms of ammonia-induced cell death in rat cortical neurons: roles of NMDA receptors and glutathione. Neurochem Int. 2005; 47:51–57. 10.1016/j.neuint.2005.04.006 15985217

[9] Yang L, Omori K, Suzukawa J, Inagaki C. Calcineurin-mediated BAD Ser155 dephosphorylation in ammonia-induced apoptosis of cultured rat hippocampal neurons. Neurosci Lett. 2004; 357:73–75. 10.1016/j.neulet.2003.12.032 15036616

[10] Felipo V, Grau E, Miñana MD, Grisolía S. Ammonium injection induces an N-methyl-D-aspartate receptor-mediated proteolysis of the microtubule-associated protein MAP-2. J Neurochem. 1993; 60:1626–30. 10.1111/j.1471-4159.1993.tb13384.x 8473887

[11] Arundine M, Tymianski M. Molecular mechanisms of calcium-dependent neurodegeneration in excitotoxicity. Cell Calcium. 2003; 34:325–37. 10.1016/S0143-4160(03)00141-6 12909079

[12] Farjam M, Beigi Zarandi FB, Farjadian S, Geramizadeh B, Nikseresht AR, Panjehshahin MR. Inhibition of NR2B-Containing N-methyl-D-Aspartate Receptors (NMDARs) in Experimental Autoimmune Encephalomyelitis, a Model of Multiple Sclerosis. Iran J Pharm Res. 2014; 13:695–705. 25237366 PMC4157046

[13] Coleman M. Axon degeneration mechanisms: commonality amid diversity. Nat Rev Neurosci. 2005; 6:889–98. 10.1038/nrn1788 16224497

[14] Kanaan NM, Pigino GF, Brady ST, Lazarov O, Binder LI, Morfini GA. Axonal degeneration in Alzheimer’s disease: when signaling abnormalities meet the axonal transport system. Exp Neurol. 2013; 246:44–53. 10.1016/j.expneurol.2012.06.003 22721767 PMC3465504

[15] Tagliaferro P, Burke RE. Retrograde Axonal Degeneration in Parkinson Disease. J Parkinsons Dis. 2016; 6:1–15. 10.3233/JPD-150769 27003783 PMC4927911

[16] Fischer-Hayes LR, Brotherton T, Glass JD. Axonal degeneration in the peripheral nervous system: implications for the pathogenesis of amyotrophic lateral sclerosis. Exp Neurol. 2013; 246:6–13. 10.1016/j.expneurol.2013.05.001 23664960

[17] Gu Y, Hamajima N, Ihara Y. Neurofibrillary tangle-associated collapsin response mediator protein-2 (CRMP-2) is highly phosphorylated on Thr-509, Ser-518, and Ser-522. Biochemistry. 2000; 39:4267–75. 10.1021/bi992323h 10757975

[18] Chung MA, Lee JE, Lee JY, Ko MJ, Lee ST, Kim HJ. Alteration of collapsin response mediator protein-2 expression in focal ischemic rat brain. Neuroreport. 2005; 16:1647–53. 10.1097/01.wnr.0000176520.49841.e6 16189471

[19] Minturn JE, Fryer HJ, Geschwind DH, Hockfield S. TOAD-64, a gene expressed early in neuronal differentiation in the rat, is related to unc-33, a C. elegans gene involved in axon outgrowth. J Neurosci. 1995; 15:6757–66. 10.1523/JNEUROSCI.15-10-06757.1995 7472434 PMC6578000

[20] Fukada M, Watakabe I, Yuasa-Kawada J, Kawachi H, Kuroiwa A, Matsuda Y, Noda M. Molecular characterization of CRMP5, a novel member of the collapsin response mediator protein family. J Biol Chem. 2000; 275:37957–65. 10.1074/jbc.M003277200 10956643

[21] Yuasa-Kawada J, Suzuki R, Kano F, Ohkawara T, Murata M, Noda M. Axonal morphogenesis controlled by antagonistic roles of two CRMP subtypes in microtubule organization. Eur J Neurosci. 2003; 17:2329–43. 10.1046/j.1460-9568.2003.02664.x 12814366

[22] Yoshimura T, Kawano Y, Arimura N, Kawabata S, Kikuchi A, Kaibuchi K. GSK-3beta regulates phosphorylation of CRMP-2 and neuronal polarity. Cell. 2005; 120:137–49. 10.1016/j.cell.2004.11.012 15652488

[23] Ip JP, Fu AK, Ip NY. CRMP2: functional roles in neural development and therapeutic potential in neurological diseases. Neuroscientist. 2014; 20:589–98; Epub ahead of print. 10.1177/1073858413514278 24402611

[24] Arimura N, Hattori A, Kimura T, Nakamuta S, Funahashi Y, Hirotsune S, Furuta K, Urano T, Toyoshima YY, Kaibuchi K. CRMP-2 directly binds to cytoplasmic dynein and interferes with its activity. J Neurochem. 2009; 111:380–90. 10.1111/j.1471-4159.2009.06317.x 19659462

[25] Rosslenbroich V, Dai L, Baader SL, Noegel AA, Gieselmann V, Kappler J. Collapsin response mediator protein-4 regulates F-actin bundling. Exp Cell Res. 2005; 310:434–44. 10.1016/j.yexcr.2005.08.005 16181627

[26] Nishimura T, Fukata Y, Kato K, Yamaguchi T, Matsuura Y, Kamiguchi H, Kaibuchi K. CRMP-2 regulates polarized Numb-mediated endocytosis for axon growth. Nat Cell Biol. 2003; 5:819–26. 10.1038/ncb1039 12942088

[27] Kawano Y, Yoshimura T, Tsuboi D, Kawabata S, Kaneko-Kawano T, Shirataki H, Takenawa T, Kaibuchi K. CRMP-2 is involved in kinesin-1-dependent transport of the Sra-1/WAVE1 complex and axon formation. Mol Cell Biol. 2005; 25:9920–35. 10.1128/MCB.25.22.9920-9935.2005 16260607 PMC1280248

[28] Kimura T, Watanabe H, Iwamatsu A, Kaibuchi K. Tubulin and CRMP-2 complex is transported via Kinesin-1. J Neurochem. 2005; 93:1371–82. 10.1111/j.1471-4159.2005.03063.x 15935053

[29] Arimura N, Ménager C, Kawano Y, Yoshimura T, Kawabata S, Hattori A, Fukata Y, Amano M, Goshima Y, Inagaki M, Morone N, Usukura J, Kaibuchi K. Phosphorylation by Rho kinase regulates CRMP-2 activity in growth cones. Mol Cell Biol. 2005; 25:9973–84. 10.1128/MCB.25.22.9973-9984.2005 16260611 PMC1280267

[30] Touma E, Kato S, Fukui K, Koike T. Calpain-mediated cleavage of collapsin response mediator protein(CRMP)-2 during neurite degeneration in mice. Eur J Neurosci. 2007; 26:3368–81. 10.1111/j.1460-9568.2007.05943.x 18052987

[31] Zhang JN, Michel U, Lenz C, Friedel CC, Köster S, d’Hedouville Z, Tönges L, Urlaub H, Bähr M, Lingor P, Koch JC. Calpain-mediated cleavage of collapsin response mediator protein-2 drives acute axonal degeneration. Sci Rep. 2016; 6:37050. 10.1038/srep37050 27845394 PMC5109185

[32] Camins A, Verdaguer E, Folch J, Pallàs M. Involvement of calpain activation in neurodegenerative processes. CNS Drug Rev. 2006; 12:135–48. 10.1111/j.1527-3458.2006.00135.x 16958987 PMC6494133

[33] Yang Z, Lin F, Robertson CS, Wang KK. Dual vulnerability of TDP-43 to calpain and caspase-3 proteolysis after neurotoxic conditions and traumatic brain injury. J Cereb Blood Flow Metab. 2014; 34:1444–52. 10.1038/jcbfm.2014.105 24917042 PMC4158661

[34] Zhang Z, Ottens AK, Sadasivan S, Kobeissy FH, Fang T, Hayes RL, Wang KK. Calpain-mediated collapsin response mediator protein-1, -2, and -4 proteolysis after neurotoxic and traumatic brain injury. J Neurotrauma. 2007; 24:460–72. 10.1089/neu.2006.0078 17402852

[35] Liu W, Zhou XW, Liu S, Hu K, Wang C, He Q, Li M. Calpain-truncated CRMP-3 and -4 contribute to potassium deprivation-induced apoptosis of cerebellar granule neurons. Proteomics. 2009; 9:3712–28. 10.1002/pmic.200800979 19639589

[36] Aylsworth A, Jiang SX, Desbois A, Hou ST. Characterization of the role of full-length CRMP3 and its calpain-cleaved product in inhibiting microtubule polymerization and neurite outgrowth. Exp Cell Res. 2009; 315:2856–68. 10.1016/j.yexcr.2009.06.014 19559021

[37] Halpin LE, Northrop NA, Yamamoto BK. Ammonia mediates methamphetamine-induced increases in glutamate and excitotoxicity. Neuropsychopharmacology. 2014; 39:1031–38. 10.1038/npp.2013.306 24165886 PMC3924538

[38] Gerencser AA, Mark KA, Hubbard AE, Divakaruni AS, Mehrabian Z, Nicholls DG, Polster BM. Real-time visualization of cytoplasmic calpain activation and calcium deregulation in acute glutamate excitotoxicity. J Neurochem. 2009; 110:990–1004. 10.1111/j.1471-4159.2009.06194.x 19493161 PMC2745075

[39] Tan M, Ma S, Huang Q, Hu K, Song B, Li M. GSK-3α/β-mediated phosphorylation of CRMP-2 regulates activity-dependent dendritic growth. J Neurochem. 2013; 125:685–97. 10.1111/jnc.12230 23470087

[40] Albrecht J, Jones EA. Hepatic encephalopathy: molecular mechanisms underlying the clinical syndrome. J Neurol Sci. 1999; 170:138–46. 10.1016/S0022-510X(99)00169-0 10617392

[41] Felipo V, Butterworth RF. Neurobiology of ammonia. Prog Neurobiol. 2002; 67:259–79. 10.1016/S0301-0082(02)00019-9 12207972

[42] Norenberg MD. Astrocytic-ammonia interactions in hepatic encephalopathy. Semin Liver Dis. 1996; 16:245–53. 10.1055/s-2007-1007237 8989810

[43] Felipo V. Hepatic encephalopathy: effects of liver failure on brain function. Nat Rev Neurosci. 2013; 14:851–58. 10.1038/nrn3587 24149188

[44] Marcaida G, Miñana MD, Burgal M, Grisolía S, Felipo V. Ammonia prevents activation of NMDA receptors by glutamate in rat cerebellar neuronal cultures. Eur J Neurosci. 1995; 7:2389–96. 10.1111/j.1460-9568.1995.tb01036.x 8845943

[45] Hermenegildo C, Montoliu C, Llansola M, Muñoz MD, Gaztelu JM, Miñana MD, Felipo V. Chronic hyperammonemia impairs the glutamate-nitric oxide-cyclic GMP pathway in cerebellar neurons in culture and in the rat in vivo. Eur J Neurosci. 1998; 10:3201–09. 10.1046/j.1460-9568.1998.00329.x 9786213

[46] Rodrigo R, Erceg S, Felipo V. Neurons exposed to ammonia reproduce the differential alteration in nitric oxide modulation of guanylate cyclase in the cerebellum and cortex of patients with liver cirrhosis. Neurobiol Dis. 2005; 19:150–61. 10.1016/j.nbd.2004.12.001 15837570

[47] Cabrera-Pastor A, Arenas YM, Taoro-Gonzalez L, Montoliu C, Felipo V. Chronic hyperammonemia alters extracellular glutamate, glutamine and GABA and membrane expression of their transporters in rat cerebellum. Modulation by extracellular cGMP. Neuropharmacology. 2019. 10.1016/j.neuropharm.2019.01.011 30641078

[48] Taoro-Gonzalez L, Arenas YM, Cabrera-Pastor A, Felipo V. Hyperammonemia alters membrane expression of GluA1 and GluA2 subunits of AMPA receptors in hippocampus by enhancing activation of the IL-1 receptor: underlying mechanisms. J Neuroinflammation. 2018; 15:36. 10.1186/s12974-018-1082-z 29422059 PMC5806265

[49] Balzano T, Dadsetan S, Forteza J, Cabrera-Pastor A, Taoro-Gonzalez L, Malaguarnera M, Gil-Perotin S, Cubas-Nuñez L, Casanova B, Castro-Quintas A, Ponce-Mora A, Arenas YM, Leone P, et al. Chronic hyperammonemia induces peripheral inflammation that leads to cognitive impairment in rats: reversal by anti-tnfa treatment. J Hepatol. 2019. 10.1016/j.jhep.2019.01.008 30654069

[50] Hassan SS, Baumgarten TJ, Ali AM, Füllenbach ND, Jördens MS, Häussinger D, Butz M, Schnitzler A, Groiss SJ. Cerebellar inhibition in hepatic encephalopathy. Clin Neurophysiol. 2019; 130:886–92. 10.1016/j.clinph.2019.02.020 30981173

[51] Norenberg MD. Oxidative and nitrosative stress in ammonia neurotoxicity. Hepatology. 2003; 37:245–48. 10.1053/jhep.2003.50087 12540772

[52] Kosenko E, Felipo V, Montoliu C, Grisolía S, Kaminsky Y. Effects of acute hyperammonemia in vivo on oxidative metabolism in nonsynaptic rat brain mitochondria. Metab Brain Dis. 1997; 12:69–82. 10.1007/BF02676355 9101539

[53] Kruczek C, Görg B, Keitel V, Pirev E, Kröncke KD, Schliess F, Häussinger D. Hypoosmotic swelling affects zinc homeostasis in cultured rat astrocytes. Glia. 2009; 57:79–92. 10.1002/glia.20737 18709649

[54] Görg B, Bidmon HJ, Häussinger D. Gene expression profiling in the cerebral cortex of patients with cirrhosis with and without hepatic encephalopathy. Hepatology. 2013; 57:2436–47. 10.1002/hep.26265 23325665

[55] Jayakumar AR, Rama Rao KV, Tong XY, Norenberg MD. Calcium in the mechanism of ammonia-induced astrocyte swelling. J Neurochem. 2009 (Suppl 1); 109:252–57. 10.1111/j.1471-4159.2009.05842.x 19393035 PMC4737088

[56] Shah NJ, Neeb H, Kircheis G, Engels P, Häussinger D, Zilles K. Quantitative cerebral water content mapping in hepatic encephalopathy. Neuroimage. 2008; 41:706–17. 10.1016/j.neuroimage.2008.02.057 18456518

[57] Bobermin LD, Wartchow KM, Flores MP, Leite MC, Quincozes-Santos A, Gonçalves CA. Ammonia-induced oxidative damage in neurons is prevented by resveratrol and lipoic acid with participation of heme oxygenase 1. Neurotoxicology. 2015; 49:28–35. 10.1016/j.neuro.2015.05.005 26003724

[58] Fukata Y, Itoh TJ, Kimura T, Ménager C, Nishimura T, Shiromizu T, Watanabe H, Inagaki N, Iwamatsu A, Hotani H, Kaibuchi K. CRMP-2 binds to tubulin heterodimers to promote microtubule assembly. Nat Cell Biol. 2002; 4:583–91. 10.1038/ncb825 12134159

[59] Sasaki Y, Cheng C, Uchida Y, Nakajima O, Ohshima T, Yagi T, Taniguchi M, Nakayama T, Kishida R, Kudo Y, Ohno S, Nakamura F, Goshima Y. Fyn and Cdk5 mediate semaphorin-3A signaling, which is involved in regulation of dendrite orientation in cerebral cortex. Neuron. 2002; 35:907–20. 10.1016/S0896-6273(02)00857-7 12372285

[60] Hou ST, Jiang SX, Aylsworth A, Ferguson G, Slinn J, Hu H, Leung T, Kappler J, Kaibuchi K. CaMKII phosphorylates collapsin response mediator protein 2 and modulates axonal damage during glutamate excitotoxicity. J Neurochem. 2009; 111:870–81. 10.1111/j.1471-4159.2009.06375.x 19735446

[61] Brittain JM, Piekarz AD, Wang Y, Kondo T, Cummins TR, Khanna R. An atypical role for collapsin response mediator protein 2 (CRMP-2) in neurotransmitter release via interaction with presynaptic voltage-gated calcium channels. J Biol Chem. 2009; 284:31375–90. 10.1074/jbc.M109.009951 19755421 PMC2781534

[62] Chi XX, Schmutzler BS, Brittain JM, Wang Y, Hingtgen CM, Nicol GD, Khanna R. Regulation of N-type voltage-gated calcium channels (Cav2.2) and transmitter release by collapsin response mediator protein-2 (CRMP-2) in sensory neurons. J Cell Sci. 2009; 122:4351–62. 10.1242/jcs.053280 19903690 PMC2779133

[63] Brittain JM, Wang Y, Eruvwetere O, Khanna R. Cdk5-mediated phosphorylation of CRMP-2 enhances its interaction with CaV2.2. FEBS Lett. 2012; 586:3813–18. 10.1016/j.febslet.2012.09.022 23022559

[64] Yu J, Moutal A, Dorame A, Bellampalli SS, Chefdeville A, Kanazawa I, Pham NY, Park KD, Weimer JM, Khanna R. Phosphorylated CRMP2 Regulates Spinal Nociceptive Neurotransmission. Mol Neurobiol. 2018. 10.1007/s12035-018-1445-6 30565051 PMC6581648

[65] Zhang J, Zhao B, Zhu X, Li J, Wu F, Li S, Gong X, Cha C, Guo G. Phosphorylation and SUMOylation of CRMP2 regulate the formation and maturation of dendritic spines. Brain Res Bull. 2018; 139:21–30. 10.1016/j.brainresbull.2018.02.004 29425794

[66] Tan M, Li Z, Ma S, Luo J, Xu S, Lu A, Gan W, Su P, Lin H, Li S, Lai B. Heroin activates Bim via c-Jun N-terminal kinase/c-Jun pathway to mediate neuronal apoptosis. Neuroscience. 2013; 233:1–8. 10.1016/j.neuroscience.2012.12.005 23262244

[67] Contestabile A. Cerebellar granule cells as a model to study mechanisms of neuronal apoptosis or survival in vivo and in vitro. Cerebellum. 2002; 1:41–55. 10.1080/147342202753203087 12879973

[68] Feinberg K, Kolaj A, Wu C, Grinshtein N, Krieger JR, Moran MF, Rubin LL, Miller FD, Kaplan DR. A neuroprotective agent that inactivates prodegenerative TrkA and preserves mitochondria. J Cell Biol. 2017; 216:3655–75. 10.1083/jcb.201705085 28877995 PMC5674898

[69] Sasaki Y, Araki T, Milbrandt J. Stimulation of nicotinamide adenine dinucleotide biosynthetic pathways delays axonal degeneration after axotomy. J Neurosci. 2006; 26:8484–91. 10.1523/JNEUROSCI.2320-06.2006 16914673 PMC6674352

[70] Zhang J, Tan M, Yin Y, Ren B, Jiang N, Guo G, Chen Y. Distinct Functions of Endophilin Isoforms in Synaptic Vesicle Endocytosis. Neural Plast. 2015; 2015:371496. 10.1155/2015/371496 26682072 PMC4670672

[71] Ji Z, Tan M, Gao Y, Zhang J, Gong X, Guo G, Lin H. CRMP-5 interacts with tubulin to promote growth cone development in neurons. Int J Clin Exp Med. 2014; 7:67–75. 24482690 PMC3902242

[72] Zhang J, Fan J, Tian Q, Song Z, Zhang JF, Chen Y. Characterization of two distinct modes of endophilin in clathrin-mediated endocytosis. Cell Signal. 2012; 24:2043–50. 10.1016/j.cellsig.2012.06.006 22750032

[73] Cha C, Zhang J, Ji Z, Tan M, Li S, Wu F, Chen K, Gong S, Guo G, Lin H. CRMP4 regulates dendritic growth and maturation via the interaction with actin cytoskeleton in cultured hippocampal neurons. Brain Res Bull. 2016; 124:286–94. 10.1016/j.brainresbull.2016.06.008 27339813

[74] Zhang J, Yin Y, Ji Z, Cai Z, Zhao B, Li J, Tan M, Guo G. Endophilin2 Interacts with GluA1 to Mediate AMPA Receptor Endocytosis Induced by Oligomeric Amyloid-β. Neural Plast. 2017; 2017:8197085. 10.1155/2017/8197085 28758034 PMC5516760

